# A longitudinal study of pre-pregnancy antioxidant levels and subsequent perinatal outcomes in black and white women: The CARDIA Study

**DOI:** 10.1371/journal.pone.0229002

**Published:** 2020-02-14

**Authors:** Emily W. Harville, Cora E. Lewis, Janet M. Catov, David R. Jacobs, Myron D. Gross, Erica P. Gunderson

**Affiliations:** 1 Department of Epidemiology, Tulane School of Public Health and Tropical Medicine, New Orleans, Los Angeles, United States of America; 2 Division of Preventive Medicine, University of Alabama-Birmingham, Birmingham, Alabama, United States of America; 3 University of Pittsburgh Departments of OB/GYN and Epidemiology, Pittsburgh, Pennsylvania, United States of America; 4 Division of Epidemiology and Community Health, School of Public Health, University of Minnesota, Minneapolis, Minnesota, United States of America; 5 Division of Research, Cardiovascular and Metabolism Section, Kaiser Permanente Northern California, Oakland, California, United States of America; Hospital de la Santa Creu i Sant Pau (Universitat Autonoma de Barcelona, CIBERBBN), SPAIN

## Abstract

**Background:**

Although protective associations between dietary antioxidants and pregnancy outcomes have been reported, randomized controlled trials of supplementation have been almost uniformly negative. A possible explanation is that supplementation during pregnancy may be too late to have a beneficial effect. Therefore, we examined the relationship between antioxidant levels prior to pregnancy and birth outcomes.

**Methods and findings:**

Serum carotenoids and tocopherols were assayed in fasting specimens at 1985–86 (baseline) and 1992–1993 (year 7) from 1,215 participants in Coronary Artery Risk Development in Young Adults (CARDIA) study. An interviewer-administered quantitative food-frequency questionnaire assessed dietary intake of antioxidants. Pregnancy outcome was self-reported at exams every 2 to 5 years. Linear and logistic regression modeling was used to assess relationships of low birthweight (LBW; <2,500 g), continuous infant birthweight, preterm birth (PTB; <37 weeks) and length of gestation with antioxidant levels adjusted for confounders, as well as interactions with age and race.

**Results:**

In adjusted models, lycopene was associated with higher odds of LBW (adjusted odds ratio for top quartile, 2.15, 95% confidence interval 1.14, 3.92) and shorter gestational age (adjusted beta coefficient -0.50 weeks). Dietary intake of antioxidants was associated with lower birthweight, while supplement use of vitamin C was associated with higher gestational age (0.41 weeks, 0.01, 0.81).

**Conclusions:**

Higher preconception antioxidant levels are not associated with better birth outcomes.

## Introduction

Nutrition is considered a key to good maternal health during pregnancy, as well as to good birth outcomes. Partly because oxidative stress has been implicated in a number of pregnancy complications [1, 2], interest in antioxidants as a possible preventive factor has grown. A number of observational studies have indicated that higher maternal antioxidant levels, measured in serum or plasma, during pregnancy are associated with better birth outcomes. For instance, higher maternal vitamin A has been associated with a lower risk of preterm labor/birth [3], and higher birth length [4]. Vitamins C and E have been associated with a lower risk of small-for-gestational-age [5] and higher birthweight and length [6]. Other antioxidants such as α-tocopherol have also been shown to be associated with higher birthweight [7], and a case-control study of spontaneous preterm birth (PTB) found that plasma concentrations above the median of α-carotene, β-carotene, α-cryptoxanthin, β-cryptoxanthin, and lycopene were associated with lower risk of PTB, while high γ-tocopherol was associated with higher risk of PTB [8]. These studies were conducted within medical care settings and for the most part on women unlikely to be actively malnourished, although the range of countries was considerable [U.S. [7], Canada [8], South Korea [6], Algeria [5], China [4], and Turkey [3]].

Although these observational studies demonstrated significant protective associations between certain antioxidants and birth outcomes, the results of randomized controlled trials of supplementation to reduce pregnancy complications have been almost uniformly disappointing. It should be noted that the randomized controlled trials primarily addressed supplement use, while the observational trials primarily examined measured levels in biological samples and self-reported dietary intake. Generally, randomized controlled trials have found no effect on small-for-gestational-age [9]. Reviews concluded that the data are insufficient to assess the effects of vitamin C on birthweight, but there is a possibility of higher risk of PTB with supplementation [10], and the data did not support a positive effect of vitamin E on any outcome [11]. The interventions in these cases were conducted in a wide variety of populations and often focused on women at high risk for pre-eclampsia, with birthweight or PTB as a secondary outcome.

A possible explanation for these discordant results is that supplementation during pregnancy is too late to have a beneficial effect. Some studies indicate a stronger effect for periconceptional or preconceptional antioxidant or multivitamin intake than for intake during pregnancy. For instance, a study of 2064 pregnant women in North Carolina found that women with dietary intakes of vitamin C below the 10^th^ percentile were associated with preterm premature rupture of membranes [12], and this association was stronger for preconception intake compared to level of intake in the 2^nd^ trimester. During the preconception period, general multivitamin use has been associated with a lower risk in pre-eclampsia [13], PTB, and preterm labor [13–15], but a higher risk of early fetal losses [16]. Multivitamin use was particularly protective in women with BMI <25, but not in overweight or obese women [14]. The periconceptional use of a multivitamin was associated with higher birthweight in black infants [17], fewer early preterm births [18], and a lower risk of SGA and PTB [14]. These studies were conducted in the U.S. or northern Europe. Preconception and continued supplementation of vitamin A and β-carotene have shown a lower risk in maternal mortality in Nepal [19]. Given these studies, it may be that the very early period is key for setting the stage for the pregnancy (e.g., for placentation), or that the woman’s health and nutrition status before pregnancy are crucial for improving pregnancy health.

These studies share the limitations of relying on women’s self-reported vitamin use during pregnancy and retrospective report of vitamin use pre-pregnancy, and no preconception studies use biomarkers to measure the antioxidant levels. Also, they generally focus on white women. In this analysis, we examine whether serum antioxidant levels, as well as diet and supplement use, prior to the pregnancy predict birthweight and gestational age. We hypothesized that higher antioxidant levels would be associated with higher birthweight and longer gestations, and that the associations may differ by maternal age at first birth and by race.

## Methods

The Coronary Artery Risk Development in Young Adults (CARDIA) Study is a multi-center, longitudinal, observational study designed to describe the development of risk factors for cardiovascular disease in young black and white men and women. In 1985–86 (year 0), 2,787 women (52% black) aged 18–30 were enrolled at the baseline exam from four geographic areas: Birmingham, Alabama; Chicago, Illinois; Minneapolis, Minnesota; and Oakland, California [20, 21]. Participants were re-examined 2, 5, 7, 10, 15 and 20 years after baseline. The overall retention rate was 72 percent of the surviving cohort 20 years later. A variety of clinical, laboratory and lifestyle measures were obtained using standardized methods at baseline and follow-up exams [22, 23]. Institutional Review Boards at each participating study center approved all study years. Written, informed consent was obtained from subjects for all study procedures. The IRB of Tulane University ruled this secondary data analysis exempt from review.

The subset for this analysis included women with antioxidant measures assessed at year 0 or 7 as part of the Young Adult Longitudinal Trends in Antioxidants ancillary study. One thousand five hundred sixty-nine women had both pregnancy and antioxidant data (flowchart in S1 Fig). Compared to women who did not report pregnancies at any time and who participated in at least one follow-up, women who reported a pregnancy had higher serum levels of β-cryptoxanthin and lower levels of γ-tocopherol (p<0.05); there was no difference in the levels of the other measures. Women who were pregnant or breastfeeding at the time of the interview/biospecimen collection (n = 15) were excluded, due to potential changes in diet, supplement use, and antioxidant and lipid metabolism during those times. The final analytic sample of 1,215 women was limited to those with valid birthweight or gestational age information on one or more singleton live births delivered after index exam; the most common reason for not being included in this analysis was not having had any post-baseline births (n = 339).

There were three sources of information about antioxidant levels: serum measurement, reported diet, and reported supplement intake. Details of the antioxidant serum analyses have been provided elsewhere [24]. Briefly, blood samples were drawn after an overnight fast. Serum obtained at the baseline exam and the year 7 exam was used to assay the carotenoids α- and β-carotene, lycopene, zeaxanthin/lutein, and β-cryptoxanthin and the tocopherols α-tocopherol and γ-tocopherol at the Molecular Epidemiology and Biomarker Research Laboratory, University of Minnesota, USA. The carotenoids and tocopherols were measured by an HPLC-based assay at Years 0 and 7. The coefficients of variation were less than 10% for all analytes and control pools, and year 0 and 7 measurements were correlated at between r = 0.4 and r = 0.7.

The CARDIA Diet History, an interviewer-administered quantitative food-frequency questionnaire designed to be a comprehensive assessment tool for habitual intake [25], identified 1609 distinct food items at baseline and year 7. Overall, its reliability and validity have been shown to be good in whites though less consistent in blacks [26]; antioxidants were not specifically assessed in the validation study. Intake of antioxidants and total energy was based on calculations from the Diet History [26], a modified version of the Western Electric dietary history; the database from the Nutrition Coordinating Center for the Multiple Risk Factor Intervention Trial and the Lipid Research Clinics was used to calculate the nutrients from this questionnaire. The A Priori Diet Quality Score was used as a measure of diet quality [27]. This measure has been shown to be associated with health outcomes, including longevity, diabetes, myocardial infarction, and biomarkers for cardiovascular disease [28]. Dietary supplements were queried as part of the Diet History: “Do you take vitamin or mineral supplements, what kind, how many, and how often?” Answers were open-ended and amounts were added to the nutrient amounts for each participant (nutrients were recorded in the database both with and without supplements). One carotenoid (α-carotene) and three tocopherols (α-, γ-, δ-tocopherol) were examined in diet, and two carotenoids (vitamins A and E) and one tocopherol (α-tocopherol) in supplements. β-carotene supplement use was not sufficiently common to be analyzed. Diet and supplement measures were analyzed separately. Serum and diet measurements were correlated at between 0.2 and 0.3 (p<0.01). Seven women in the sample were missing information on either diet or supplement use.

Pregnancy outcomes were based on the woman’s self-report for each pregnancy. At each follow-up exam, women were asked whether they had been pregnant since the previous exam; how the pregnancy had ended; the baby’s birthweight; and length of gestation. The outcomes examined were birthweight, gestational age, and birthweight-for-gestational-age (z-score, limited to gestational ages between 32 and 43 weeks). Each outcome was examined as a continuous variable and as a dichotomous variable: low birthweight (LBW) defined as birth weight <2500 g, and PTB defined as gestational age at birth <37 weeks. To estimate growth restriction, term LBW (LBW among babies delivered after 37 weeks’ gestation) and birthweight were also examined. A validation study of maternal report of gestational age at delivery among a subset of 211 CARDIA women using medical record abstractions has been conducted [29]. Sensitivity for preterm birth <34 weeks was 100%; specificity was 99%. Sensitivity was 67% and specificity was 89% for preterm births delivered at 34–36 weeks. The overall sensitivity for maternal report of ever delivering preterm (<37 weeks) was 84% (16/19), and the specificity was 89% (170/192). We conducted additional analysis of term low birthweight (to minimize the effect of the correlation of gestational age and birthweight); the results were similar and did not appear to add more information beyond the birthweight and gestational age analyses. A supplementary analysis of macrosomia (birthweight >4000 g) was also conducted. The first pregnancy after the first available antioxidant measure was used in analyses.

Analyses were conducted both considering the predictors as quartiles and continuous variables. Supplement intake was highly right-skewed; it was examined as both a log-transformed continuous variable and yes/no for any supplement intake (few enough women reported supplement use for more detailed categories to be useful). Multiple linear and logistic modeling was used to adjust for the a priori selected potential confounders of smoking (current/former/never), race (black/white), age at included pregnancy, BMI (continuous), education (highest degree based on years of school at the follow-up prior to the pregnancy), parity (0, 1, 2+), physical activity (total physical activity intensity score (quartiles) [30], diet quality, and marital status(married/not). Interactions with age, race, smoking, parity, and BMI were examined using a product term in the model; 3-way interactions were also checked. Age at included pregnancy interactions were examined both as continuous and dichotomous variables; for ease of presentation, dichotomous results are provided (age ≤30 and > 30).

We also examined an interaction with time (both continuous and dichotomized at 2 years), to determine whether any associations differed if the measures were taken closer in time to the pregnancy. No interactions were found. Standardizing antioxidant levels for lipids is recommended in some circumstances [24], but lipid levels could also be an intermediate between antioxidant intake and birth outcomes [31, 32], in which case this adjustment could bias the results. However, adjustment for lipids made no difference to the results.

## Results

Mean age at included pregnancy was 30.3 years. Women were evenly split between black or white races. Sixty-three and five-tenths percent had a pre-pregnancy BMI in the normal range; 62.2% were never-smokers, and 45.5% were nulliparous at their CARDIA index exam. Eight and three-tenths percent of the first infants born after the antioxidant measurement were low birthweight and 18.3% born preterm (Table 1). Carotenoid levels were on average higher in white women than black, and in women older than 30, although no differences were found in lycopene levels.

**Table 1:** Description of study population at their CARDIA index exam in 1985–86 or 1992–93, women with antioxidant measures and subsequent singleton livebirth (n = 1215).

	Black (n = 601)					White (n = 614)				
	N	%				N	%			
age at pregnancy										
18-<25	142	23.7				33	5.4			
25-<30	209	34.8				174	28.3			
30-<35	180	30.0				276	45.0			
≥35	69	11.5				131	21.3			
Parity										
0	227	37.8				326	53.1			
1	173	28.8				178	29.0			
2+	201	33.4				110	17.9			
Smoking at time of pregnancy										
nonsmoker	399	66.8				352	57.6			
former smoker	43	7.2				125	20.5			
smoker	155	26.0				134	21.9			
BMI category										
15–18.5	37	6.2				35	5.7			
18.5–25	309	51.9				457	74.8			
>25–30	118	19.8				84	13.8			
>30	132	22.2				35	5.7			
low birthweight (1st singleton livebirth after antioxidant measure)	67	12.0				25	4.5			
preterm birth (1st singleton livebirth after antioxidant measure)	146	24.4				76	12.4			
baseline	mean	median	std	p25	p75	mean	median	std	p25	p75
alpha carotene (mg/dl)	1.8	1.3	1.6	0.8	2.1	4.2	3.1	4.1	1.8	5.0
beta carotene (mg/dl)	14.2	11.3	11.4	7.5	18.1	19.5	15.3	15.2	10.0	23.7
lutein/zeaxanthine (mg/dl)	17.7	16.6	7.4	12.6	21.7	19.3	17.7	9.4	12.4	24.1
beta cryptoxanthine (mg/dl)	8.4	7.0	5.1	5.1	10.4	9.3	7.8	6.3	5.2	11.5
alpha tocopherol	0.8	0.8	0.2	0.7	0.9	1.0	1.0	0.2	0.8	1.1
gamma tocopherol	0.2	0.2	0.1	0.1	0.3	0.2	0.2	0.1	0.1	0.2
lycopene (mg/dl)	29.8	28.4	14.6	19.1	38.1	28.6	27.1	14.0	17.5	37.7
birthweight (1st singleton livebirth after baseline)	3202	3260	699	2920	3628	3454	3459	545	3147	3799
gestational age (1st singleton livebirth after baseline)	38.5	40.0	3.5	37.0	40.0	39.3	40	2.5	38.0	41.0
time between measurement and pregnancy (years)	3.9	3.4	2.6	1.8	5.4	4.3	3.9	2.7	2.2	5.9

P<0.01 for all race comparisons

BMI, body mass index

### Serum antioxidant levels and birth outcomes

Among the carotenoids (Table 2), in unadjusted analysis, no associations were found with low birthweight, except for lycopene (unadjusted odds ratio (OR) for highest vs. lowest quartile 2.40, 95% confidence intervals (CI) 1.31,4.37, adjusted OR (aOR) 2.15, 1.14,3.92). β-carotene was associated with a higher risk of preterm birth (aOR 1.62, 1.01, 2.26); the third quartile of β-cryptoxanthin was also associated with a higher risk (aOR 1.73, 1.11, 2.71). In unadjusted analysis (Table 3) α-carotene was found to be associated with higher birthweight (beta coefficient 158 g, 95% CI 51,265 for the highest quartile) and birthweight for gestational age, while lycopene was found to be associated with lower birthweight (-130 g, 95% CI -235, -24); both results were reduced after adjustment. Lycopene was found to be associated with shorter gestational age (adjusted beta coefficient -0.50 weeks for the highest quartile, -0.99, -0.01). Among the tocopherols, only α-tocopherol (119 g, 31, 225) was found to be associated with higher birthweight, in unadjusted analyses only. Detailed examination of the results suggested that race, smoking, and pre-pregnancy BMI were the strongest confounders (changed the effect estimate the most).

**Table 2:** Relationship between serum antioxidant status, as quartiles, and subsequent birth outcome.

	low birthweight (n = 1114)				preterm birth (n = 1206)			
	unadjusted		adjusted^a^		unadjusted		adjusted^a^	
	OR	95% CI	OR	95% CI	OR	95% CI	OR	95% CI
carotenoids								
α-carotene (μg/dl)^b^								
<1.2	1.0		1.0		1.0		1.0	
1.2–2.1	0.83	(0.47, 1.46)	1.09	(0.61, 1.95)	0.84	(0.57, 1.25)	1.01	(0.67, 1.51)
2.1–4.0	0.67	(0.37, 1.21)	0.93	(0.47, 1.84)	0.75	(0.51, 1.12)	1.42	(0.90, 2.23)
4.0–32.1	0.63	(0.34, 1.17)	1.02	(0.46, 2.27)	0.49	(0.32, 0.76)	0.96	(0.54, 1.69)
β-carotene (μg/dl)								
<8.6	1.0		1.0		1.0		1.0	
8.6–13.6	0.70	(0.39, 1.25)	0.78	(0.42, 1.44)	1.20	(0.80, 1.81)	1.41	(0.92, 2.17)
13.6–22.7	0.68	(0.38, 1.23)	0.99	(0.53, 1.84)	1.23	(0.82, 1.85)	1.64	(1.05, 2.56)
22.7–167.2	0.72	(0.40, 1.29)	0.94	(0.48, 1.85)	0.89	(0.58, 1.36)	1.62	(1.01, 2.62)
lutein/zeaxanthin (μg/dl)								
<12.7	1.0		1.0		1.0		1.0	
12.7–17.0	0.70	(0.37, 1.32)	0.71	(0.37, 1.36)	0.89	(0.60, 1.34)	0.96	(0.63, 1.46)
17.1–23.6	1.06	(0.59, 1.88)	1.04	(0.57, 1.89)	0.99	(0.67, 1.48)	1.09	(0.72, 1.65)
23.7–58.5	0.94	(0.94, 1.70)	0.99	(0.52, 1.88)	0.72	(0.47, 1.10)	0.82	(0.52, 1.30)
β-cryptoxanthin (μg/dl)								
<5.2	1.0		1.0		1.0		1.0	
5.2–7.2	0.67	(0.36, 1.27)	0.63	(0.32, 1.24)	1.39	(0.91, 2.11)	1.46	(0.94, 2.27)
7.2–10.7	0.81	(0.44, 1.49)	0.85	(0.44, 1.62)	1.46	(0.96, 2.22)	1.73	(1.11, 2.71)
10.7–51.4	1.27	(0.73, 2.23)	1.47	(0.78, 2.77)	1.16	(0.75, 1.79)	1.50	(0.93, 2.40)
Lycopene (μg/dl)								
<19.7	1.0		1.0		1.0		1.0	
19.7–29.2	1.21	(0.62, 2.37)	1.11	(0.56, 2.20)	1.34	(0.82, 1.89)	1.39	(0.90, 2.14)
29.2–39.1	1.07	(0.54, 2.12)	1.11	(0.56, 2.22)	0.95	(0.62, 1.47)	0.94	(0.60, 1.49)
39.2–91.4	2.40	(1.31, 4.37)	2.15	(1.14, 3.92)	1.48	(0.98, 2.23)	1.50	(0.98, 2.30)
tocopherols								
α-tocopherol (mg/dl)								
<0.77	1.0		1.0		1.0		1.0	
0.77–0.90	0.89	(0.50, 1.58)	0.75	(0.41, 1.36)	0.98	(0.66,1.47)	1.05	(0.69, 1.58)
0.90–1.05	1.05	(0.60, 1.83)	0.94	(0.53, 1.69)	0.93	(0.62,1.39)	1.20	(0.79, 1.83)
1.06–2.22	0.47	(0.24, 0.94)	0.64	(0.32, 1.29)	0.70	(0.46, 1.07)	1.08	(0.68, 1.72)
γ-tocopherol (mg/dl)								
<0.14	1.0		1.0		1.0		1.0	
0.14–0.19	1.54	(0.81, 2.90)	1.60	(0.84, 3.08)	1.07	(0.69, 1.65)	0.92	(0.59, 1.44)
0.19–0.24	1.48	(0.78, 2.82)	1.35	(0.68, 2.65)	1.39	(0.92, 2.11)	1.04	(0.67, 1.61)
0.24–0.55	1.52	(0.80, 2.88)	1.43	(0.73, 2.79)	1.40	(0.92, 2.13)	1.15	(0.74, 1.77)

OR, odds ratio; CI, confidence interval; BMI, body mass index

^a^ Antioxidants are categorized into quartiles. Adjusted models adjusted for age at pregnancy, BMI, diet quality, education, parity, physical activity, race, smoking, marital status

^b^ Each antioxidant is categorized into quartiles

**Table 3:** Relationship between serum antioxidant status, as quartiles, and subsequent birth outcome.

	birthweight				gestational age				birthweight for gestational age (z-score)			
	unadjusted		adjusted^a^		unadjusted		adjusted^a^		unadjusted		adjusted^a^	
	β	95% CI	β	95% CI	β	95% CI	β	95% CI	β	95% CI	β	95% CI
carotenoids^b^												
α-carotene (μg/dl)												
<1.2	0		0		0		0		0		0	
1.2–2.1	91	(-14, 196)	-7	(-112, 98)	-0.05	(-0.55, 0.44)	-0.29	(-0.79, 0.20)	0.25	(0.08, 0.42)	0.17	(0.00, 0.34)
2.1–4.0	92	(-13, 198)	-52	(-167, 64)	-0.17	(-0.66, 0.33)	-0.79	(-1.34, -0.24)	0.27	(0.10, 0.44)	0.13	(-0.06, 0.31)
4.0–32.1	158	(51, 265)	-92	(-226, 42)	0.34	(-0.15, 0.84)	-0.54	(-1.17, 0.08)	0.25	(0.08, 0.42)	-0.02	(-0.23, 0.20)
β-carotene (μg/dl)												
<8.6	0		0		0		0		0		0	
8.6–13.6	83	(-22, 188)	38	(-65, 142)	-0.01	(-0.50, 0.48)	-0.16	(-0.65, 0.33)	0.18	(0.01, 0.35)	0.10	(-0.07, 0.27)
13.6–22.7	48	(-57, 154)	-43	(-151, 65)	-0.45	(-0.95, 0.04)	-0.71	(-1.22, -0.20)	0.26	(0.09, 0.43)	0.12	(-0.06, 0.29)
22.7–167.2	84	(-22, 190)	-31	(-145, 82)	-0.01	(-0.51, 0.48)	-0.63	(-1.17, -0.09)	0.18	(0.01, 0.35)	0.10	(-0.08, 0.28)
lutein/zeaxanthin (μg/dl)												
<12.7	0		0		0		0		0		0	
12.7–17.0	36	(-69, 142)	43	(-62, 148)	-0.15	(-0.65, 0.34)	-0.20	(-0.70, 0.29)	0.16	(0.00, 0.33)	0.19	(0.02, 0.36)
17.1–23.6	24	(-83, 130)	22	(-84, 128)	0.00	(-0.49, 0.50)	-0.12	(-0.62, 0.38)	0.12	(0.09, 0.29)	0.14	(-0.03, 0.31)
23.7–58.5	-0.30	(-107, 106)	-26	(-136, 85)	0.24	(-0.26, 0.73)	0.10	(-0.42, 0.62)	0.06	(0.01, 0.23)	0.04	(-0.14, 0.21)
β-cryptoxanthin (μg/dl)												
<5.2	0		0		0		0		0		0	
5.2–7.2	37	(-69, 143)	34	(-70, 138)	-0.29	(-0.78, 0.21)	-0.30	(-0.79, 0.20)	0.16	(-0.01, 0.32)	0.15	(-0.01, 0.32)
7.2–10.7	32	(-74, 138)	2	(-106, 110)	-0.33	(-0.82, 0.17)	-0.51	(-1.02, -0.00)	0.17	(0.00, 0.34)	0.17	(-0.01, 0.34)
10.7–51.4	-22	(-128, 85)	-72	(-184, 40)	-0.23	(-0.72, 0.27)	-0.46	(-0.99, 0.06)	0.07	(-0.10, 0.24)	0.03	(-0.15, 0.21)
Lycopene (μg/dl)												
<19.7	0		0		0		0		0		0	
19.7–29.2	-52	(-158, 54)	-21	(-125, 83)	-0.17	(-0.66, 0.32)	-0.16	(-0.65, 0.33)	-0.05	(-0.22, 0.12)	0.00	(-0.16, 0.17)
29.2–39.1	24	(-82, 129)	20	(-84, 123)	0.00	(-0.49, 0.50)	-0.01	(-0.50, 0.48)	0.05	(-0.12, 0.22)	0.05	(-0.12, 0.21)
39.2–91.4	-130	(-235, -24)	-89	(-193, 14)	-0.58	(-1.07, -0.09)	-0.50	(-0.99, -0.01)	-0.05	(-0.22, 0.12)	0.00	(-0.17, 0.17)
tocopherols												
α-tocopherol (mg/dl)												
<0.77	0		0		0		0		0		0	
0.77–0.90	-27	(-132, 78)	-14	(-118, 90)	-0.29	(-0.78, 0.20)	-0.23	(-0.72, 0.26)	0.08	(-0.09, 0.24)	0.03	(-0.14, 0.19)
0.90–1.05	72	(-33, 177)	29	(-77, 135)	-0.19	(-0.68, 0.30)	-0.32	(-0.82, 0.18)	0.29	(0.12, 0.45)	0.22	(0.05, 0.39)
1.06–2.22	119	(13, 225)	13	(-98, 124)	0.38	(-0.11, 0.87)	0.06	(-0.46, 0.59)	0.21	(0.04, 0.38)	0.06	(-0.11, 0.24)
γ-tocopherol (mg/dl)												
<0.14	0		0		0		0		0		0	
0.14–0.19	-29	(-134, 76)	-37	(-140, 66)	-0.13	(-0.62, 0.36)	-0.15	(-0.64, 0.34)	0.06	(-0.11, 0.23)	0.01	(-0.15, 0.18)
0.19–0.24	-67	(-173, 40)	-21	(-126, 84)	-0.37	(-0.86, 0.13)	-0.16	(-0.65, 0.33)	0.05	(-0.12, 0.22)	0.07	(-0.10, 0.24)
0.24–0.55	-56	(-162, 50)	-34	(-141, 72)	-0.40	(-0.89, 0.09)	-0.24	(-0.74, 0.27)	0.03	(-162, 0.20)	0.03	(-0.14, 0.20)

CI, confidence interval; BMI, body mass index

^a^Antioxidants are categorized into quartiles. Adjusted models adjusted for age at pregnancy, BMI, diet quality, education, parity, physical activity, race, smoking, marital status

^b^ Each antioxidant is categorized into quartiles

### Interaction analysis

Significant interactions were found between age at included pregnancy and many of the serum antioxidants. Among younger (<30 years) women, higher carotenoids were associated with higher risk of LBW, while these patterns were not seen in older women (Table 4); for PTB, this pattern was seen for β-carotene. Further examination of 3-way interactions (age, race and antioxidants) found that the higher risk of LBW associated with carotenoids was largely limited to the younger black women, while among the older black women, these factors were neutral or protective (p for 3-way interaction ≤ 0.05; S1 Table). Similar patterns, though not as strong, held for gestational age (Table 5).

**Table 4:** Relationship between serum antioxidant status, as quartiles, and subsequent birth outcome, stratified by age.

	low birthweight					preterm birth				
	adjusted, age = younger^a^, n = 531		adjusted, age = older, n = 583			adjusted, age = younger, n = 556		adjusted, age = older, n = 656		
	OR	95% CI	OR	95% CI	p for interaction	OR	95% CI	OR	95% CI	p for interaction
carotenoids^b^										
α-carotene (μg/dl)					<0.01					0.59
<1.2	1.0		1.0			1.0		1.0		
1.2–2.1	1.32	(0.60, 2.91)	0.84	(0.61, 1.95)		0.87	(0.53, 1.44)	1.55	(0.74, 3.23)	
2.1–4.0	1.55	(0.57, 4.20)	0.51	(0.47, 1.84)		1.09	(0.59, 2.02)	2.13	(1.03, 4.42)	
4.0–32.1	5.18	(1.50, 17.89)	0.36	(0.46, 2.27)		1.35	(0.58, 3.14)	1.02	(0.44, 2.37)	
β-carotene (μg/dl)					<0.01					0.17
<8.6	1.0		1.0			1.0		1.0		
8.6–13.6	2.04	(0.82, 5.05)	0.26	(0.42, 1.44)		1.15	(0.66, 1.99)	1.82	(1.82, 3.71)	
13.6–22.7	2.41	(0.90, 6.42)	0.40	(0.53, 1.84)		1.40	(0.77, 2.54)	1.89	(1.89, 3.85)	
22.7–167.2	4.08	(1.53, 10.94)	0.20	(0.48, 1.85)		2.13	(1.14, 3.98)	1.25	(1.25, 2.72)	
lutein/zeaxanthin (μg/dl)					0.01					0.67
<12.7	1.0		1.0			1.0		1.0		
12.7–17.0	1.00	(0.38, 2.64)	0.50	(0.37, 1.36)		1.18	(0.68, 2.04)	0.71	(0.71, 1.41)	
17.1–23.6	1.60	(0.65, 3.93)	0.68	(0.57, 1.89)		1.12	(0.64, 1.95)	1.05	(1.05, 1.99)	
23.7–58.5	2.73	(1.08, 6.91)	0.38	(0.52, 1.88)		0.96	(0.50, 1.84)	0.71	(0.71, 1.38)	
β-cryptoxanthin (μg/dl)					0.05					0.39
<5.2	1.0		1.0			1.0		1.0		
5.2–7.2	1.21	(0.47, 3.12)	0.30	(0.32, 1.24)		1.85	(1.04, 3.27)	1.01	(1.01, 2.02)	
7.2–10.7	1.93	(0.75, 4.96)	0.34	(0.44, 1.62)		2.09	(1.15, 3.79)	1.17	(1.17, 2.33)	
10.7–51.4	2.97	(1.14, 7.77)	0.68	(0.78, 2.77)		1.71	(0.90, 3.26)	1.14	(1.14, 2.34)	
Lycopene (μg/dl)					0.02					0.48
<19.7										
19.7–29.2	0.58	(0.18, 1.94)	1.55	(0.41, 1.36)		1.26	(0.69, 2.32)	1.49	(0.79, 2.80)	
29.2–39.1	1.07	(0.38, 2.99)	1.15	(0.53, 1.69)		0.75	(0.40,1.40)	1.13	(0.57, 2.23)	
39.2–91.4	3.34	(1.35, 8.24)	1.26	(0.32, 1.29)		1.73	(0.96, 3.10)	1.16	(0.61, 2.22)	
tocopherols										
α-tocopherol (mg/dl)					0.02					0.48
<0.77	1.0		1.0			1.0		1.0		
0.77–0.90	1.40	(0.60, 3.29)	0.39	(0.84, 3.08)		0.97	(0.58, 1.63)	1.18	(0.59, 2.38)	
0.90–1.05	2.25	(0.95, 5.36)	0.40	(0.68, 2.65)		1.02	(0.58, 1.80)	1.43	(0.73, 2.78)	
1.06–2.22	1.96	(0.67, 5.74)	0.27	(0.73, 2.79)		1.27	(0.66, 2.44)	1.02	(0.51, 2.04)	
γ-tocopherol (mg/dl)					<0.01					0.66
<0.14	1.0		1.0			1.0		1.0		
0.14–0.19	2.18	(0.79, 5.95)	1.26	(0.56, 2.20)		0.86	(0.46, 1.58)	1.04	(1.04, 2.00)	
0.19–0.24	1.63	(0.55, 4.80)	1.17	(0.56, 2.22)		1.22	(0.67, 2.25)	0.86	(0.86, 1.65)	
0.24–0.55	2.11	(0.76, 5.85)	1.02	(1.14, 3.92)		1.25	(0.96, 2.27)	0.99	(0.99, 1.89)	

OR, odds ratio; CI, confidence interval; BMI, body mass index

^a^adjusted for age at pregnancy, BMI, diet quality, education, parity, physical activity, race, smoke, marital status; Younger: ≤30 yrs, older:>30

^b^ Each antioxidant is categorized into quartiles

**Table 5:** Relationship between serum antioxidant status, as quartiles, and subsequent birth outcome, stratified by age.

	birthweight					gestational age					birthweight-for-gestational-age				
	adjusted, age = younger^a^, n = 531		adjusted, age = older, n = 583			adjusted, age = younger, n = 556		adjusted, age = older, n = 656			adjusted, age = younger^a^, n = 498		adjusted, age = older, n = 563		
	β	95% CI	β	95% CI		β	95% CI	β	95% CI		β	95% CI	β	95% CI	
carotenoids															
α-carotene (μg/dl)					0.12					0.04					0.49
<1.2	0		0			0		0			0		0		
1.2–2.1	46	(-87, 179)	-104	(-273, 65)		-0.14	(-0.82, 0.55)	-0.61	(-1.34, 0.12)		0.22	(0.01, 0.42)	0.11	(-0.17, 0.40)	
2.1–4.0	-139	(-301, 23)	-2	(-167, 170)		-0.78	(-1.62, 0.05)	-0.88	(-1.61, -0.15)		0.01	(-0.24, 0.26)	0.22	(-0.06, 0.51)	
4.0–32.1	-293	(-511, -75)	-16	(-198, 166)		-1.30	(-2.42, -0.18)	-0.35	(-1.13, 0.43)		-0.06	(-0.40, 0.28)	0.05	(-0.25, 0.36)	
β-carotene (μg/dl)					0.33					<0.01					0.02
<8.6	0		0			0		0			0		0		
8.6–13.6	22	(-119, 162)	82	(-73, 237)		-0.07	(-0.79, 0.64)	-0.22	(-0.89, 0.46)		0.08	(-0.14, 0.29)	0.15	(-0.11, 0.41)	
13.6–22.7	-29	(-186, 129)	-30	(-182, 122)		-0.87	(-1.67, 0.08)	-0.45	(-1.11, 0.22)		0.22	(-0.02, 0.47)	0.04	(-0.21, 0.30)	
22.7–167.2	-136	(-302, 31)	61	(-98, 219)		-1.49	(-2.34, -0.64)	-0.03	(-0.72, 0.65)		0.24	(-0.02, 0.49)	0.01	(-0.26, 0.27)	
lutein/zeaxanthin (μg/dl)					0.55					0.42					0.14
<12.7	0		0			0		0							
12.7–17.0	58	(-88, 203)	25	(-128, 178)		-0.32	(-1.07, 0.42)	-0.07	(-0.73, 0.60)		0		0		
17.1–23.6	64	(-85, 212)	-22	(-173, 129)		-0.17	(-0.93, 0.59)	-0.09	(-0.75, 0.57)		0.30	(0.08, 0.52)	0.07	(-0.18, 0.33)	
23.7–58.5	-120	(-289, 49)	22	(-125, 170)		-0.13	(-1.00, 0.74)	0.21	(-0.43, 0.85)		0.23	(0.01, 0.46)	0.03	(-0.23, 0.28)	
β-cryptoxanthin (μg/dl)					0.71					0.09					0.18
<5.2	0		0			0		0			0		0		
5.2–7.2	-4	(-148, 140)	90	(-61, 241)		-0.59	(-1.32, 0.15)	0.00	(-0.66, 0.66)		0.11	(-0.11, 0.33)	0.23	(-0.02, 0.48)	
7.2–10.7	-37	(-190, 117)	53	(-100, 206)		-0.90	(-1.68, -0.13)	-0.03	(-0.69, 0.64)		0.21	(-0.02, 0.44)	0.15	(-0.10, 0.41)	
10.7–51.4	-119	(-282, 44)	-14	(-170, 142)		-0.81	(-1.63, 0.02)	-0.14	(-0.81, 0.54)		0.05	(-0.20, 0.30)	0.04	(-0.22, 0.30)	
Lycopene (μg/dl)					0.11					0.09					0.66
<19.7	0		0			0		0			0		0		
19.7–29.2	32	(-124, 188)	-71	(-210, 68)		0.21	(-0.59, 1.01)	-0.42	(-1.02, 0.18)		-0.05	(-0.29, 0.19)	0.03	(-0.20, 0.26)	
29.2–39.1	30	(-122, 183)	3	(-141, 146)		0.29	(-0.49, 1.07)	-0.24	(-0.86, 0.38)		0.08	(-0.15, 0.32)	-0.02	(-0.26, 0.22)	
39.2–91.4	-169	(-326, -13)	28	(-166, 110)		-0.94	(-1.73, -0.14)	-0.01	(-0.61, 0.59)		0.02	(-0.22, 0.26)	-0.04	(-0.27, 0.19)	
tocopherols															
α-tocopherol (mg/dl)					0.13					0.50					0.38
<0.77	0		0			0		0			0		0		
0.77–0.90	-76	(-214, 63)	72	(-85, 229)		-0.13	(-0.83, 0.58)	-0.27	(-0.95, 0.41)		-0.10	(-0.32, 0.11)	0.19	(-0.07, 0.45)	
0.90–1.05	-48	(-196, 100)	126	(-25, 278)		-0.42	(-1.18, 0.33)	-0.25	(-0.91, 0.41)		0.13	(-0.10, 0.36)	0.34	(0.08, 0.59)	
1.06–2.22	-86	(-259, 87)	108	(-43, 258)		0.13	(-.75, 1.02)	-0.02	(-0.67, 0.64)		-0.14	(-0.40, 0.13)	0.24	(-0.01, 0.49)	
γ-tocopherol (mg/dl)					0.17					0.93					0.04
<0.14	0		0			0		0			0		0		
0.14–0.19	-50	(-204, 104)	-22	(-162, 118)		0.11	(-0.67, 0.90)	-0.35	(-0.97, 0.26)		-0.06	(-0.29, 0.17)	0.08	(-0.16, 0.31)	
0.19–0.24	-23	(-183, 136)	-13	(-152, 126)		-0.35	(-1.16, 0.46)	0.00	(-0.60, 0.60)		0.10	(-0.14, 0.34)	0.05	(-0.18, 0.28)	
0.24–0.55	34	(-120, 189)	-106	(-253, 41)		-0.15	(-0.95, 0.64)	-0.20	(-0.83, 0.43)		0.21	(-0.03, 0.45)	-0.15	(-0.39, 0.10)	

CI, confidence interval; BMI, body mass index

^a^adjusted for age at pregnancy, BMI, diet quality, education, parity, physical activity, race, smoke, marital status; Younger: ≤30 yrs, older:>30

^b^ Each antioxidant is categorized into quartiles

### Dietary antioxidant intake and birth outcomes

Stronger associations for diet were seen for birthweight than dichotomous outcomes(Table 6), with β-carotene, α-tocopherol, γ-tocopherol, and δ-tocopherol all associated with lower birthweight (adjusted beta coefficients for highest quartile, -120 g, -132 g, -136 g, -177 g, respectively, all *P*<0.05; Table 7).

**Table 6:** Relationship between dietary intake of antioxidants and subsequent birth outcomes.

	low birthweight (n = 1111)		preterm birth (n = 1206)	
	OR^a^	95% CI	OR^a^	95% CI
carotenoids				
β-carotene (mcg)				
<1702	1.0		1.0	
1,702–2,888	1.07	(0.57, 2.02)	0.90	(0.60, 1.36)
2,888–5,061	0.94	(0.47, 1.87)	0.80	(0.52, 1.24)
5,071–86,529	1.64	(0.83, 3.24)	0.95	(0.59, 1.52)
Tocopherols				
α-tocopherol				
<5.49	1.0		1.0	
5.49–7.76	1.73	(0.87, 3.41)	1.32	(0.86, 2.01)
7.77–11.40	1.25	(0.62, 2.54)	0.92	(0.59, 1.43)
11.42–106.07	1.82	(0.94, 3.53)	1.16	(0.76, 1.77)
γ-tocopherol				
<10.58	1.0		1.0	
10.58–15.63	1.26	(0.62, 2.59)	1.45	(0.92, 2.28)
15.74–22.79	1.65	(0.84, 3.26)	1.46	(0.94, 2.28)
22.81–99.81	1.80	(0.93, 3.51)	1.40	(0.90, 2.19)
δ-tocopherol				
<3.40	1.0		1.0	
3.40–5.18	1.51	(0.67, 3.41)	1.23	(0.73, 2.07)
5.19–7.78	1.34	(0.59, 3.06)	1.23	(0.73, 2.08)
7.80–33.95	2.12	(0.97, 4.63)	1.34	(0.79, 2.26)

^a^adjusted for age at pregnancy, BMI, education, parity, physical activity, race, smoking, marital status, diet quality

^b^ Each antioxidant is categorized into quartiles.

**Table 7:** Relationship between dietary intake of antioxidants and subsequent birth outcomes.

	Birthweight (n = 1111)		gestational age (n = 1206)		Birthweight-for-gestational-age (n = 1058)	
	β*	95% CI	β*	95% CI	β*	95% CI
carotenoids^b^						
β-carotene (mcg)						
<17.2	0		0		0	
1,702–2,888	8	(-95, 111)	0.11	(-0.38, 0.61)	0.02	(-0.14, 0.19)
2,888–5,061	50	(-58, 159)	0.27	(-0.25, 0.79)	0.10	(-0.07, 0.28)
5,071–86,529	-120	(-239, -1)	-0.17	(-0.74, 0.39)	-0.15	(-0.34, 0.04)
tocopherols						
α-tocopherol						
<5.49	0		0		0	
5.49–7.76	-24	(-127, 79)	-0.17	(-0.66, 0.32)	-0.01	(-0.18, 0.15)
7.77–11.40	-40	(-143, 64)	0.12	(-0.37, 0.61)	-0.09	(-0.26, 0.08)
11.42–106.07	-132	(-236, -28)	-0.12	(-0.62, 0.37)	-0.14	(-0.31, 0.03)
γ-tocopherol						
<10.58	0		0		0	
10.58–15.63	-1	(-104, 101)	-0.38	(-0.87, 0.11)	0.00	(-0.17, 0.16)
15.74–22.79	-104	(-208, -1)	-0.46	(-0.95, 0.03)	-0.06	(-0.23, 0.11)
22.81–99.81	-136	(-241, -30)	-0.47	(-0.97, 0.03)	-0.14	(-0.31, 0.04)
δ-tocopherol						
<3.40	0		0		0	
3.40–5.18	-69	(-189, 51)	-0.29	(-0.89, 0.30)	-0.07	(-0.25, 0.12)
5.19–7.78	-75	(-196, 47)	-0.40	(-1.01, 0.20)	-0.05	(-0.24, 0.14)
7.80–33.95	-177	(-304, -51)	-0.48	(-1.10, 0.15)	-0.17	(-0.37, 0.04)

^a^adjusted for age at pregnancy, BMI, education, parity, physical activity, race, smoking, marital status, diet quality

^b^ Each antioxidant is categorized into quartiles.

### Supplement antioxidant intake and birth outcomes

Intake of vitamin C and α-tocopherol were associated with higher birthweight, but adjustment for confounders attenuated these associations (S2 Table). Gestational age was higher with estimated intake (adjusted beta coefficient 0.06, 0.01–0.12) and any supplement intake of vitamin C (0.41 weeks, 0.01–0.81). Any intake of α-tocopherol supplements was associated with a lower odds of LBW (adjusted OR 0.38, 0.19–0.76). Unlike serum antioxidant levels, there were no patterns of interaction between age at first pregnancy and dietary or supplement intake, and once age and race were accounted for, no interactions were found with smoking.

All results were similar when exposures were considered as continuous variables, and/or adjusted for lipids (supplementary material). No strong or consistent associations were found with macrosomia (S3 Table).

## Discussion

In this study, we did not find significant support for the idea that preconception antioxidant levels reduce the risk of preterm birth or low birthweight. Most protective associations with serum or dietary antioxidants that we found were eliminated by adjustment for confounders, while a few associations indicating that higher antioxidants were associated with worse birth outcomes were found. On the other hand, supplement use of vitamin C and tocopherols was associated with lower risk of low birthweight (in some analyses), perhaps suggesting confounding by health-consciousness, self-care, or other nutrients included in multivitamins. This is consistent with the randomized trials that have found that supplementation during pregnancy does not improve birth outcomes [9, 33], and may even worsen them [34–36]. The most extensive previous study of antioxidant levels during pregnancy, a study of 812 white nulliparous English women, found higher serum lutein was associated with higher risk of preterm premature rupture of membranes, but α-carotene, β-carotene, cryptoxanthin, lycopene, retinol, α-tocopherol, and γ-tocopherol were not [37]. Higher plasma γ-tocopherol was also associated with higher preterm birth risk in one study [8], while serum β-cryptoxanthin was associated with history of preterm birth in NHANES [38], so our results are broadly consistent with previous studies incorporating biomarkers.

One notable finding was the fairly consistent interaction with age at pregnancy. Generally, the negative effects were found most strongly in younger women, and protective effects were largely limited to older women. This might indicate that effects of aging were somewhat offset by antioxidant use, but among younger women, negative effects predominated. This trend was particularly pronounced in Black women. A few studies have found race differences in antioxidant relationships with metabolism: lower β-carotene has been associated with insulin resistance [39] and oxidative stress has been associated with lower insulin sensitivity in African-Americans [40], but these are health-protective, rather than the negative effect we found. Research also suggests that multivitamin use may be particularly protective against low birthweight in Black women [17]. Although we did not find an interaction with BMI, and all results are adjusted for BMI, it is possible that differences in BMI or patterns of weight gain are also affecting metabolism. Age and race are both strong predictors of BMI, and there may be residual confounding.

Antioxidants have been hypothesized to improve birth outcomes by protecting the placental membranes from damage due to reactive oxygen species [41]. Oxidative DNA damage has been associated with worse fetal growth [42], and oxidative stress with lower birthweight and gestational length [43]. However, our results do not support that hypothesis. The association of blood tocopherols and lycopene with birth outcomes may be a reflection of consuming diets relatively high in fat and low in other nutrients. Such a preconception dietary pattern could be maintained during pregnancy, influencing development of the fetus and contributing to low birthweight. The dietary pattern can be associated with blood lycopene and tocopherols due to an increase in their absorption with high-fat diets; lycopene and tocopherols are the most lipophilic compounds of the antioxidants examined. Lycopene and tocopherols are not always indicators of fruit and vegetable intakes as are other carotenoids, but have been associated with meat intake in some situations [44]. The most common sources of lycopene, in particular, in the U. S. diet, are tomato products such as pizza sauce, which may not be associated with other positive nutritional behaviors [45]. This association may be partially influenced by blood lipids and adjustment for blood lipids had a small effect on the relationship (supplementary materials). A study in London found that South Asian vegetarian women had shorter duration of gestation and lower birthweights [46], but a larger study of preconception diet found no relationship between birth outcomes and vegetarian diet [47]. Generally, both meat-eating and vegetarian diets are considered adequate for pregnant women [48]. Thus, the overall quality of the diet may have a larger effect than the antioxidant effect of the lycopene and tocopherols.

Strengths of the study were the prospective design, including measures prior to pregnancy; biracial sample, and the biomarkers of antioxidant status; both diet and supplement intake as well as serum levels could vary considerably over the time period studied, which would likely reduce study power. Weaknesses include the variable time period between the antioxidant measurements and the pregnancy. Another consideration is the self-report of gestational age, which may have biased the findings toward the null; the proportion of preterm births is on the high side, suggesting a degree of measurement error. Measurement of nutritional intake is notoriously prone to error, and although we attempted to adjust for overall diet quality, such measures can be no more than an estimate. In addition, nutritional intakes covary, and factors such as folic acid, known to be associated with pregnancy health and to affect antioxidant metabolism [49], were not considered. However, as such factors are generally associated with better health, they would be more likely to create a spurious positive association than mask one. The large number of statistical tests makes multiple comparisons an issue, although results were fairly consistent across outcomes and within classes of nutrient. In addition, our study was conducted in the United States, and the women were not likely to be actively malnourished. Results might be different in a developing country or in particularly deprived populations, or in other race/ethnic groups.

## Conclusions

Higher preconception antioxidant levels were not associated with better birth outcomes, and results were more consistent with worsened outcomes for some indicators in this sample.

## Supporting information

S1 Fig (DOCX)

S1 Table Relationship between antioxidant status (continuous z-score) and subsequent birth outcomes, interaction with age and race. (DOCX)

S2 Table Relationship between antioxidant intake from supplements and subsequent birth outcome. (DOCX)

S3 Table Associations between antioxidant measures and macrosomia (n = 1112). (DOCX)

## Abbreviations

LBW: low birthweight
PTB: preterm birth
SGA: small-for-gestational-age
CARDIA: Coronary Artery Risk Development in Young Adults
OR: odds ratio
CI: confidence interval
BMI: body mass index
