# Supplementary material for: A longitudinal study of pre-pregnancy antioxidant levels and subsequent perinatal outcomes in black and white women: The CARDIA Study
Source: PLoS One. 2020 Feb 14;15(2):e0229002. doi: 10.1371/journal.pone.0229002 (PMC7021312; doi:10.1371/journal.pone.0229002)
Supplement: S1 Fig — (DOCX) [file pone.0229002.s001.docx]

2787 women with initial exam

1638 with information on pregnancy

1569 with pregnancy and antioxidant data

1149 with no reported pregnancies or no valid pregnancy data after baseline

69 with no antioxidant data

339 no valid data on birthweight or gestational age for any singleton livebirths

1215 for analysis

15 pregnant or breastfeeding at time of interview/biospecimen collection
