# Supplementary material for: A longitudinal study of pre-pregnancy antioxidant levels and subsequent perinatal outcomes in black and white women: The CARDIA Study
Source: PLoS One. 2020 Feb 14;15(2):e0229002. doi: 10.1371/journal.pone.0229002 (PMC7021312; doi:10.1371/journal.pone.0229002)
Supplement: S1 Table — (DOCX) [file pone.0229002.s002.docx]

| Table S1. Relationship between antioxidant status (continuous z-score) and subsequent birth outcomes, interaction with age and race | | | | | | | | | | | | | | | | |
| --- | --- | --- | --- | --- | --- | --- | --- | --- | --- | --- | --- | --- | --- | --- | --- | --- |
|  |  | low birthweight | | | | | | | | | | | | | | |
|  |  | adjusted,^a^ age=younger | | | | | | |  | adjusted,^a^ age=older | | | | | | |
|  |  | race=black (n=335) | | |  | race=white (n=196) | | |  | race=black (n=221) | | |  | race=white (n=362) | | |
|  |  | OR | 95% CI | |  | OR | 95% CI | |  | OR | 95% CI | |  | OR | 95% CI | |
| carotenoids |  |  |  | |  |  |  | |  |  |  | |  |  |  | |
| α-carotene |  | 3.08 | (1.26, 7.53) | |  | 1.47 | (0.79, 2.72) | |  | 0.25 | (0.07, 0.87) | |  | 1.06 | (0.69, 1.64) | |
| β-carotene |  | 2.36 | (1.43, 3.91) | |  | 1.14 | (0.49, 2.66) | |  | 0.22 | (0.09, 0.58) | |  | 1.04 | (0.65, 1.67) | |
| lutein/zeaxanthin |  | 1.47 | (0.98, 2.20) | |  | 1.38 | (0.67, 2.83) | |  | 0.57 | (0.34, 0.98) | |  | 1.06 | (0.64, 1.76) | |
| β-cryptoxanthin |  | 1.52 | (1.00, 2.30) | |  | 1.03 | (0.45, 2.37) | |  | 0.65 | (0.36, 1.17) | |  | 1.35 | (0.96, 1.91) | |
| lycopene |  | 2.20 | (1.53, 3.16) | |  | 1.53 | (0.77, 3.06) | |  | 0.72 | (0.47, 1.09) | |  | 1.54 | (0.91, 2.59) | |
| tocopherols |  |  |  | |  |  |  | |  |  |  | |  |  |  | |
| α-tocopherol |  | 1.43 | (0.87, 2.35) | |  | 1.42 | (0.57, 3.52) | |  | 0.44 | (0.23, 0.84) | |  | 0.84 | (0.47, 1.49) | |
| γ-tocopherol |  | 1.38 | (0.88, 2.15) | |  | 0.95 | (0.41, 2.21) | |  | 1.18 | (0.76, 1.82) | |  | 1.09 | (0.58, 2.04) | |
|  |  |  |  |  |  |  |  |  |  |  |  |  |  |  |  |  |
|  |  | preterm birth | | | | | | | | | | | | | | |
|  |  | adjusted, age=younger | | | | | | |  | adjusted, age=older | | | | | | |
|  |  | race=black (n=349) | | |  | race=white (n=207) | | |  | race=black (n=249) | | |  | race=white (n=407) | | |
|  |  | OR | 95% CI | |  | OR | 95% CI | |  | OR | 95% CI | |  | OR | 95% CI | |
| carotenoids |  |  |  | |  |  |  | |  |  |  | |  |  |  | |
| α-carotene |  | 1.52 | (0.79, 2.94) | |  | 1.32 | (0.88, 1.99) | |  | 1.17 | (0.65, 2.11) | |  | 0.88 | (0.63, 1.24) | |
| β-carotene |  | 1.65 | (1.14, 2.39) | |  | 1.27 | (0.84, 1.91) | |  | 1.24 | (0.92, 1.68) | |  | 1.05 | (0.77, 1.43) | |
| lutein/zeaxanthin |  | 0.91 | (0.66, 1.24) | |  | 0.97 | (0.61, 1.53) | |  | 0.90 | (0.63, 1.27) | |  | 0.95 | (0.67, 1.34) | |
| β-cryptoxanthin |  | 1.04 | (0.75, 1.44) | |  | 1.25 | (0.80, 1.93) | |  | 0.88 | (0.60, 1.28) | |  | 1.13 | (0.86, 1.49) | |
| lycopene |  | 1.32 | (1.03, 1.69) | |  | 1.11 | (0.73, 1.69) | |  | 0.95 | (0.70, 1.30) | |  | 1.09 | (0.77, 1.54) | |
| tocopherols |  |  |  | |  |  |  | |  |  |  | |  |  |  | |
| α-tocopherol |  | 1.27 | (0.90, 1.80) | |  | 0.93 | (0.56, 1.54) | |  | 1.24 | (0.85, 1.80) | |  | 0.95 | (0.66, 1.37) | |
| γ-tocopherol |  | 1.04 | (0.76, 1.42) | |  | 1.35 | (0.86, 2.13) | |  | 1.08 | (0.76, 1.52) | |  | 1.00 | (0.66, 1.51) | |

|  |  | birthweight | | | | | | | | | | | | | | | | | | | | | |
| --- | --- | --- | --- | --- | --- | --- | --- | --- | --- | --- | --- | --- | --- | --- | --- | --- | --- | --- | --- | --- | --- | --- | --- |
|  |  | adjusted, age=younger | | | | | | | | | |  | adjusted, age=older | | | | | | | | | | |
|  |  | race=black (n=335) | | |  | | race=white (n=196) | | | | |  | race=black (n=221) | | | |  | | race=white (n=362) | | | | |
|  |  | β | 95% CI | |  | | β | | 95% CI | | |  | β | | 95% CI | |  | | β | | | 95% CI | |
| carotenoids |  |  |  | |  | |  | |  | | |  |  | |  | |  | |  | | |  | |
| α-carotene |  | -270 | (-463, -77) | |  | | -55 | | (-138, 28) | | |  | 153 | | (-38, 343) | |  | | -10 | | | (-60, 40) | |
| β-carotene |  | -147 | (-258, -36) | |  | | -11 | | (-101, 80) | | |  | 7 | | (-89, 103) | |  | | -15 | | | (-67, 36) | |
| lutein/zeaxanthin |  | -52 | (-141, 37) | |  | | -36 | | (-129, 57) | | |  | -27 | | (-138, 84) | |  | | 8 | | | (-46, 62) | |
| β-cryptoxanthin |  | -42 | (-137, 53) | |  | | -5 | | (-98, 88) | | |  | 26 | | (-95, 146) | |  | | -35 | | | (-82, 12) | |
| lycopene |  | -103 | (-175, -31) | |  | | 3 | | (-81, 87) | | |  | 67 | | (-31, 165) | |  | | -23 | | | (-79, 33) | |
| tocopherols |  |  |  | |  | |  | |  | | |  |  | |  | |  | |  | | |  | |
| α-tocopherol |  | -30 | (-133, 73) | |  | | -33 | | (-135, 70) | | |  | 42 | | (-80, 164) | |  | | 6 | | | (-51, 63) | |
| γ-tocopherol |  | 4 | (-87, 94) | |  | | 76 | | (-18, 171) | | |  | -99 | | (-210, 11) | |  | | -34 | | | (-103, 35) | |
|  |  |  |  |  | |  | |  | |  |  | | |  |  |  | |  | |  |  | |  |
|  |  | gestational age | | | | | | | | | | | | | | | | | | | | | |
|  |  | adjusted, age=younger | | | | | | | | | |  | adjusted, age=older | | | | | | | | | | |
|  |  | race=black (n=349) | | |  | | race=white (n=207) | | | | |  | race=black (n=249) | | | |  | | race=white (n=407) | | | | |
|  |  | β | 95% CI | |  | | β | | 95% CI | | |  | β | | 95% CI | |  | | β | | | 95% CI | |
| carotenoids |  |  |  | |  | |  | |  | | |  |  | |  | |  | |  | | |  | |
| α-carotene |  | -1.48 | (-2.49, -0.46) | |  | | -0.31 | | (-0.72, 0.09) | | |  | -0.09 | | (-0.94, 0.77) | |  | | 0.10 | | | (-0.10, 0.30) | |
| β-carotene |  | -1.25 | (-1.83, -0.68) | |  | | -0.28 | | (-0.72, 0.16) | | |  | -0.19 | | (-0.63, 0.25) | |  | | 0.03 | | | (-0.18, 0.25) | |
| lutein/zeaxanthin |  | -0.14 | (-0.60, 0.33) | |  | | 0.19 | | (-0.27, 0.64) | | |  | -0.03 | | (-0.51, 0.45) | |  | | 0.12 | | | (-0.11, 0.35) | |
| β-cryptoxanthin |  | -0.35 | (-0.84, 0.15) | |  | | -0.16 | | (-0.61, 0.30) | | |  | 0.11 | | (-0.40, 0.63) | |  | | -0.14 | | | (-0.34, 0.06) | |
| lycopene |  | -0.51 | (-0.89-0.14) | |  | | -0.19 | | (-0.60, 0.22) | | |  | 0.24 | | (-0.19, 0.67) | |  | | -0.11 | | | (-0.35, 0.13) | |
| tocopherols |  |  |  | |  | |  | |  | | |  |  | |  | |  | |  | | |  | |
| α-tocopherol |  | -0.32 | (-0.86, 0.23) | |  | | 0.41 | | (-0.08, 0.90) | | |  | -0.14 | | (-0.69, 0.41) | |  | | -0.05 | | | (-0.30, 0.19) | |
| γ-tocopherol |  | -0.03 | (-0.51, 0.44) | |  | | 0.06 | | (-0.41, 0.53) | | |  | -0.21 | | (-0.70, 0.27) | |  | | -0.02 | | | (-0.31, 0.28) | |
| OR, odds ratio; CI, confidence interval; BMI, body mass index | | | | | | | | | | | | | | | | | | | | | | | |
| ^a^adjusted for age at pregnancy, BMI, diet quality, education, parity, physical activity, race, smoke, marital status | | | | | | | | | | | | | | | | | | | | | | | |
