# Supplementary material for: A longitudinal study of pre-pregnancy antioxidant levels and subsequent perinatal outcomes in black and white women: The CARDIA Study
Source: PLoS One. 2020 Feb 14;15(2):e0229002. doi: 10.1371/journal.pone.0229002 (PMC7021312; doi:10.1371/journal.pone.0229002)
Supplement: S2 Table — (DOCX) [file pone.0229002.s003.docx]

| Table S2. Relationship between antioxidant intake from supplements and subsequent birth outcome | | | | | | | | | |
| --- | --- | --- | --- | --- | --- | --- | --- | --- | --- |
| **absolute levels** |  | low birthweight (n=1111) | | | | | | | |
|  |  | unadjusted | |  | adjusted^a^ | |  | adjusted^b^ | |
|  |  | OR | 95% CI |  | OR | 95% CI |  | OR | 95% CI |
| carotenoids |  |  |  |  |  |  |  |  |  |
| vitamin A |  | 0.95 | (0.89, 1.00) |  | 0.96 | (0.90, 1.02) |  | 0.96 | (0.91, 1.02) |
| vitamin C |  | 0.88 | (0.80, 0.95) |  | 0.89 | (0.81, 0.97) |  | 0.90 | (0.82, 0.98) |
| tocopherol |  |  |  |  |  |  |  |  |  |
| α-tocopherol |  | 0.80 | (0.69, 0.93) |  | 0.81 | (0.69, 0.94) |  | 0.82 | (0.70, 0.95) |
|  |  | birthweight | | | | | | | |
|  |  | unadjusted | |  | adjusted^a^ | |  | adjusted^b^ | |
|  |  | β | 95% CI |  | β | 95% CI |  | β | 95% CI |
| carotenoids |  |  |  |  |  |  |  |  |  |
| vitamin A |  | 8 | (-0.23, 16) |  | 1 | (-7, 10) |  | 1 | (-8, 9) |
| vitamin C |  | 19 | (7, 30) |  | 7 | (-5, 19) |  | 7 | (-5, 19) |
| tocopherols |  |  |  |  |  |  |  |  |  |
| α-tocopherol |  | 27 | (9, 45) |  | 15 | (-3, 33) |  | 14 | (-4, 32) |
|  |  |  |  |  |  |  |  |  | |
| **any supplement use/none** |  | low birthweight | | | | | | | |
|  |  | unadjusted | |  | adjusted^a^ | |  | adjusted^b^ | |
|  |  | OR | 95% CI |  | OR | 95% CI |  | OR | 95% CI |
| carotenoids |  |  |  |  |  |  |  |  |  |
| vitamin A |  | 0.54 | (0.30, 0.97) |  | 0.62 | (0.33, 1.14) |  | 0.64 | (0.34, 1.20) |
| vitamin C |  | 0.42 | (0.24, 0.75) |  | 0.48 | (0.26, 0.87) |  | 0.50 | (0.27, 0.91) |
| tocopherols |  |  |  |  |  |  |  |  |  |
| α-tocopherol |  | 0.36 | (0.18, 0.71) |  | 0.38 | (0.19, 0.76) |  | 0.40 | (0.20, 0.81) |
|  |  | birthweight | | | | | | | |
|  |  | unadjusted | |  | adjusted^a^ | |  | adjusted^b^ | |
|  |  | β | 95% CI |  | β | 95% CI |  | β | 95% CI |
| carotenoids |  |  |  |  |  |  |  |  |  |
| vitamin A |  | 89 | (2, 176) |  | 14 | (-0.75, 104) |  | 12 | (-78, 101) |
| vitamin C |  | 126 | (45, 207) |  | 46 | (-39, 130) |  | 43 | (-42, 127) |
| tocopherol |  |  |  |  |  |  |  |  |  |
| α-tocopherol |  | 129 | (42, 216) |  | 64 | (-25, 153) |  | 60 | (-30, 149) |
| ^a^adjusted for age at pregnancy, BMI, diet quality, education, parity, physical activity, race, smoke, marital status | | | | | | | | |  |
| ^b^adjusted for age at pregnancy, BMI, diet quality, education, parity, physical activity, race, smoke, marital status, triglycerides, total cholesterol, and HDL-c. | | | | | | | | | |

| **absolute levels** |  | preterm birth (n=1206) | | | | | | | | | |
| --- | --- | --- | --- | --- | --- | --- | --- | --- | --- | --- | --- |
|  |  | unadjusted | |  | adjusted^a^ | | | |  | adjusted^b^ | |
|  |  | OR | 95% CI |  | OR | 95% CI | | |  | OR | 95% CI |
| carotenoids |  |  |  |  |  |  | | |  |  |  |
| vitamin A |  | 0.98 | (0.95, 1.01) |  | 1.01 | (0.97, 1.04) | | |  | 1.01 | (0.97, 1.04) |
| vitamin C |  | 0.93 | (0.89, 0.98) |  | 0.97 | (0.92, 1.02) | | |  | 0.97 | (0.92, 1.02) |
| tocopherol |  |  |  |  |  |  | | |  |  |  |
| α-tocopherol |  | 0.95 | (0.89, 1.03) |  | 1.00 | (0.93, 1.08) | | |  | 1.00 | (0.92, 1.08) |
|  |  | gestational age | | | | | | | | | |
|  |  | unadjusted | |  | adjusted^a^ | | | |  | adjusted^b^ | |
|  |  | β | 95% CI |  | β | | 95% CI | |  | β | 95% CI |
| carotenoids |  |  |  |  |  | |  | |  |  |  |
| vitamin A |  | 0.04 | (0.00, 0.08) |  | 0.02 | | (-0.02, 0.06) | |  | 0.02 | (-0.02, 0.06) |
| vitamin C |  | 0.09 | (0.04, 0.15) |  | 0.06 | | (0.01, 0.12) | |  | 0.06 | (0.00, 0.12) |
| tocopherols |  |  |  |  |  | |  | |  |  |  |
| α-tocopherol |  | 0.11 | (0.02, 0.19) |  | 0.07 | | (-0.02, 0.15) | |  | 0.07 | (-0.02, 0.15) |
|  |  | birthweight for gestational age | | | | | | | | | |
|  |  | unadjusted | |  | adjusted^a^ | | | |  | adjusted^b^ | |
|  |  | β | 95% CI |  | β | | 95% CI | |  | β | 95% CI |
| carotenoids |  |  |  |  |  | |  | |  |  |  |
| vitamin A |  | 0.01 | (-0.01, 0.22) |  | -0.01 | | (-0.02, 0.01) | |  | 0.00 | (-0.02, 0.01) |
| vitamin C |  | 0.02 | (0.00, 0.39) |  | 0.01 | | (-0.01, 0.02) | |  | 0.01 | (-0.01, 0.02) |
| tocopherols |  |  |  |  |  | |  | |  |  |  |
| α-tocopherol |  | 0.35 | (0.01, 0.06) |  | 0.02 | | (-0.01, 0.05) | |  | 0.02 | (-0.01, 0.05) |
|  |  |  |  |  |  | | |  |  |  |  |
| **any supplement use/none** |  | preterm birth | | | | | | | | | |
|  |  | unadjusted | |  | adjusted^a^ | | | |  | adjusted^b^ | |
|  |  | OR | 95% CI |  | OR | 95% CI | | |  | OR | 95% CI |
| carotenoids |  |  |  |  |  |  | | |  |  |  |
| vitamin A |  | 0.82 | (0.58, 1.16) |  | 1.09 | (0.75, 1.59) | | |  | 1.08 | (0.74, 1.57) |
| vitamin C |  | 0.67 | (0.48, 0.93) |  | 0.87 | (0.60, 1.25) | | |  | 0.86 | (0.60, 1.23) |
| tocopherols |  |  |  |  |  |  | | |  |  |  |
| α-tocopherol |  | 0.78 | (0.55, 1.11) |  | 1.02 | (0.70, 1.49) | | |  | 1.01 | (0.69, 1.48) |
|  |  | gestational age | | | | | | | | | |
|  |  | unadjusted | |  | adjusted^a^ | | | |  | adjusted^b^ | |
|  |  | β | 95% CI |  | β | | 95% CI | |  | β | 95% CI |
| carotenoids |  |  |  |  |  | |  | |  |  |  |
| vitamin A |  | 0.43 | (0.02, 0.83) |  | 0.20 | | (-0.22, 0.62) | |  | 0.20 | (-0.22, 0.62) |
| vitamin C |  | 0.63 | (0.25, 1.00) |  | 0.41 | | (0.01, 0.81) | |  | 0.41 | (0.01, 0.81) |
| tocopherol |  |  |  |  |  | |  | |  |  |  |
| α-tocopherol |  | 0.56 | (0.16, 0.96) |  | 0.36 | | (-0.06, 0.78) | |  | 0.36 | (-0.06, 0.78) |
|  |  | birthweight for gestational age | | | | | | | | | |
|  |  | unadjusted | |  | adjusted^a^ | | | |  | adjusted^b^ | |
|  |  | β | 95% CI |  | β | | 95% CI | |  | β | 95% CI |
| carotenoids |  |  |  |  |  | |  | |  |  |  |
| vitamin A |  | 0.13 | (0.00, 0.26) |  | 0.03 | | (-0.11, 0.16) | |  | 0.03 | (-0.11, 0.16) |
| vitamin C |  | 0.09 | (-0.05, 0.23) |  | -0.01 | | (-0.16, 0.13) | |  | -0.02 | (-0.16, 0.13) |
| tocopherol |  |  |  |  |  | |  | |  |  |  |
| α-tocopherol |  | 0.15 | (0.01, 0.29) |  | 0.06 | | (-0.08, 0.21) | |  | 0.06 | (-0.08, 0.21) |
| ^a^adjusted for age at pregnancy, BMI, diet quality, education, parity, physical activity, race, smoke, marital status | | | | | | | | | | | |
| ^b^adjusted for age at pregnancy, BMI, diet quality, education, parity, physical activity, race, smoke, marital status, triglycerides, total cholesterol, and HDL-c. | | | | | | | | | | | |
