# Supplementary material for: A longitudinal study of pre-pregnancy antioxidant levels and subsequent perinatal outcomes in black and white women: The CARDIA Study
Source: PLoS One. 2020 Feb 14;15(2):e0229002. doi: 10.1371/journal.pone.0229002 (PMC7021312; doi:10.1371/journal.pone.0229002)
Supplement: S3 Table — (DOCX) [file pone.0229002.s004.docx]

|  | | | |  | | |  | |  |  |  |  |
| --- | --- | --- | --- | --- | --- | --- | --- | --- | --- | --- | --- | --- |
| Table S3. Associations between antioxidant measures and macrosomia (n=1112) | | | | | | | | | | |  |  |
| serum antioxidants | | | | | | | | | | |  |  |
|  |  | unadjusted | | |  | adjusted^a^ | | | | |  |  |
|  |  | OR | 95% CI | |  | OR | | 95% CI | | |  |  |
| carotenoids |  |  |  | |  |  | |  | | |  |  |
| α-carotene (μg/dl) |  |  |  | |  |  | |  | | |  |  |
| <1.2 |  | 1.0 |  | |  | 1.0 | |  | | |  |  |
| 1.2-2.1 |  | 1.82 | (1.06, 3.14) | |  | 1.37 | | (0.79, 2.38) | | |  |  |
| 2.1-4.0 |  | 1.29 | (0.73, 2.29) | |  | 0.77 | | (0.41, 1.45) | | |  |  |
| 4.0-32.1 |  | 1.65 | (0.94, 2.87) | |  | 0.78 | | (0.39, 1.57) | | |  |  |
|  |  |  |  | |  |  | |  | | |  |  |
| β-carotene (μg/dl) |  |  |  | |  |  | |  | | |  |  |
| <8.6 |  | 1.0 |  | |  | 1.0 | |  | | |  |  |
| 8.6-13.6 |  | 1.47 | (0.87, 2.49) | |  | 0.89 | | (0.77, 2.26) | | |  |  |
| 13.6-22.7 |  | 1.15 | (0.66, 2.00) | |  | 1.06 | | (0.55, 1.79) | | |  |  |
| 22.7-167.2 |  | 1.33 | (0.78, 2.28) | |  | 0.95 | | (0.61, 2.03) | | |  |  |
|  |  |  |  | |  |  | |  | | |  |  |
| lutein/zeaxanthin (μg/dl) |  |  |  | |  |  | |  | | |  |  |
| <12.7 |  | 1.0 |  | |  | 1.0 | |  | | |  |  |
| 12.7-17.0 |  | 1.11 | (0.66, 1.86) | |  | 1.42 | | (0.83, 2.43) | | |  |  |
| 17.1-23.6 |  | 1.05 | (0.62, 1.77) | |  | 1.24 | | (0.71, 2.17) | | |  |  |
| 23.7-58.5 |  | 0.96 | (0.56, 1.63) | |  | 1.01 | | (0.55, 1.83) | | |  |  |
|  |  |  |  | |  |  | |  | | |  |  |
| β-cryptoxanthin (μg/dl) |  |  |  | |  |  | |  | | |  |  |
| <5.2 |  | 1.0 |  | |  | 1.0 | |  | | |  |  |
| 5.2-7.2 |  | 0.88 | (0.52, 1.50) | |  | 0.89 | | (0.52, 1.54) | | |  |  |
| 7.2-10.7 |  | 1.03 | (0.62, 1.72) | |  | 1.06 | | (0.61, 1.84) | | |  |  |
| 10.7-51.4 |  | 0.97 | (0.56, 1.63) | |  | 0.95 | | (0.54, 1.69) | | |  |  |
|  |  |  |  | |  |  | |  | | |  |  |
| Lycopene (μg/dl) |  |  |  | |  |  | |  | | |  |  |
| <19.7 |  | 1.0 |  | |  | 1.0 | |  | | |  |  |
| 19.7-29.2 |  | 0.84 | (0.48, 1.45) | |  | 0.94 | | (0.54, 1.63) | | |  |  |
| 29.2-39.1 |  | 1.46 | (0.89, 2.40) | |  | 1.48 | | (0.88, 2.48) | | |  |  |
| 39.2-91.4 |  | 0.84 | (0.48, 1.45) | |  | 0.99 | | (0.57, 1.72) | | |  |  |
|  |  |  |  | |  |  | |  | | |  |  |
| tocopherols |  |  |  | |  |  | |  | | |  |  |
| α-tocopherol (mg/dl) |  |  |  | |  |  | |  | | |  |  |
| <0.77 |  | 1.0 |  | |  | 1.0 | |  | | |  |  |
| 0.77-0.90 |  | 1.57 | (0.58, 1.75) | |  | 1.06 | | (0.61, 1.84) | | |  |  |
| 0.90-1.05 |  | 1.26 | (0.77, 2.20) | |  | 0.94 | | (0.53, 1.65) | | |  |  |
| 1.06-2.22 |  | 1.57 | (0.79, 2.26) | |  | 0.96 | | (0.54, 1.70) | | |  |  |
|  |  |  |  | |  |  | |  | | |  |  |
| γ-tocopherol (mg/dl) |  |  |  | |  |  | |  | | |  |  |
| <0.14 |  | 1.0 |  | |  | 1.0 | |  | | |  |  |
| 0.14-0.19 |  | 1.96 | (0.91, 2.71) | |  | 1.27 | | (0.73, 2.19) | | |  |  |
| 0.19-0.24 |  | 1.70 | (0.71, 2.22) | |  | 1.30 | | (0.75, 2.25) | | |  |  |
| 0.24-0.55 |  | 1.33 | (0.91, 2.72) | |  | 1.33 | | (0.77, 2.33) | | |  |  |
| OR, odds ratio; CI, confidence interval  ^a^ adjusted for age at pregnancy, BMI, diet quality, education, parity, physical activity, race, smoke, marital status  ^b^ Each antioxidant is categorized into quartiles. | | | | | | | | | | |  |  |

| dietary antioxidants | | | |  | supplements | | |
| --- | --- | --- | --- | --- | --- | --- | --- |
|  |  | OR^a^ | 95% CI |  |  | aOR | 95% CI |
| carotenoids |  |  |  |  | carotenoids |  |  |
| β-carotene (mcg) |  |  |  |  | vitamin A | 1.01 | 0.97, 1.05 |
| <1702 |  | 1.0 |  |  | vitamin C | 1.03 | 0.98, 1.10 |
| 1,702-2,888 |  | 1.31 | (0.75, 2.29) |  | tocopherol |  |  |
| 2,888-5,061 |  | 1.30 | (0.73, 2.32) |  | α-tocopherol | 1.00 | 0.91, 1.09 |
| 5,071-86,529 |  | 0.85 | (0.44, 1.64) |  |  |  |  |
| Tocopherols |  |  |  |  | carotenoids |  |  |
| α-tocopherol |  |  |  |  | vitamin A | 1.19 | 0.78, 1.80 |
| <5.49 |  | 1.0 |  |  | vitamin C | 1.04 | 0.67, 1.61 |
| 5.49-7.76 |  | 0.80 | (0.48, 1.31) |  | tocopherols |  |  |
| 7.77-11.40 |  | 0.67 | (0.40, 1.13) |  | α-tocopherol | 0.96 | 0.61, 1.49 |
| 11.42-106.07 |  | 0.60 | (0.35, 1.04) |  |  |  |  |
|  |  |  |  |  |  |  |  |
| γ-tocopherol |  |  |  |  |  |  |  |
| <10.58 |  | 1.0 |  |  |  |  |  |
| 10.58-15.63 |  | 1.14 | (0.70, 1.85) |  |  |  |  |
| 15.74-22.79 |  | 0.75 | (0.44, 1.28) |  |  |  |  |
| 22.81-99.81 |  | 0.63 | (0.35, 1.13) |  |  |  |  |
|  |  |  |  |  |  |  |  |
| δ-tocopherol |  |  |  |  |  |  |  |
| <3.40 |  | 1.0 |  |  |  |  |  |
| 3.40-5.18 |  | 1.11 | (0.63, 1.96) |  |  |  |  |
| 5.19-7.78 |  | 0.83 | (0.45, 1.54) |  |  |  |  |
| 7.80-33.95 |  | 0.66 | (0.33, 1.32) |  |  |  |  |
|  |  |  |  |  |  |  |  |
